# Supplementary material for: Multimodal sensorimotor assessment of hand and forearm asymmetries: a reliability and correlational study
Source: PeerJ. 2024 May 28;12:e17403. doi: 10.7717/peerj.17403 (PMC11141550; doi:10.7717/peerj.17403)
Supplement: Supplemental Information 1 — N=30. Values are represented as Pearsons’s ρ (level of significance). aRoM: active range of motion; ECRB: extensor carpi radialis brevis; PPT: Pressure Pain Threshold. Values in bold represent a significant correlation after correction for multiple comparisons (P<0.006). [file peerj-12-17403-s001.docx]

| **S-Table 1.** Correlation values and significance levels between different test assessing the wrist extensors muscles at the dominant side on assessment-1. | | | | | | | | | | |
| --- | --- | --- | --- | --- | --- | --- | --- | --- | --- | --- |
|  | Myoton: frequency | Myoton: stiffness | Myoton: decrement ^a^ | Nine-Hole Peg test | PPT: Deltoideus | PPT: ECRB | aRoM: extension | aRoM: flexion | aRoM: total | Isometric strength |
| Myoton: frequency | - | **0.889**  **(<0.001)** | -0.004  (0.984) | -0.206  (0.274) | 0.093  (0.624) | -0.165  (0.385) | -0.102  (0.593) | -0.244  (0.193) | -0.243  (0.196) | 0.429  (0.018) |
| Myoton: stiffness | - | - | 0.366  (0.047) | -0.163  (0.390) | 0.092  (0.628) | -0.272  (0.146) | -0.051  (0.788) | 0.048  (0.802) | -0.006  (0.974) | 0.196  (0.300) |
| Myoton: decrement | - | - | - | 0.096  (0.614) | 0.034  (0.858) | -0.350  (0.058) | 0.098  (0.605) | **0.529**  **(0.003)** | 0.423  (0.020) | -0.437  (0.016) |
| Nine-Hole Peg test | - | - | - | - | -0.215  (0.255) | -0.228 (0.226) | -0.423  (0.020) | 0.057  (0.763) | -0.206  (0.274) | -0.129  (0.497) |
| PPT: Deltoideus | - | - | - | - | - | **0.520**  **(0.003)** | 0.170  (0.369) | -0.027  (0.886) | 0.077  (0.687) | 0.239  (0.203) |
| PPT: ECRB | - | - | - | - | - | - | 0.019  (0.922) | -0.025  (0.896) | -0.010  (0.958) | 0.125  (0.511) |
| aRoM: extension | - | - | - | - | - | - | - | 0.166  (0.380) | **0.710**  **(<0.001)** | -0.350  (0.058) |
| aRoM: flexion | - | - | - | - | - | - | - | - | **0.811**  **(<0.001)** | **-0.622**  **(<0.001)** |
| aRoM: total | - | - | - | - | - | - | - | - | - | **-0.648**  **(<0.001)** |
| Isometric strength | - | - | - | - | - | - | - | - | - | - |
| N=30. Values are represented as Pearsons's ρ (level of significance). aRoM: active range of motion; ECRB: extensor carpi radialis brevis; PPT: Pressure Pain Threshold. Values in bold represent a significant correlation after correction for multiple comparisons (P<0.006). | | | | | | | | | | |

| **S-Table 2.** Correlation values and significance levels between different test assessing the wrist extensors muscles at the dominant side on assessment-2. | | | | | | | | | | |
| --- | --- | --- | --- | --- | --- | --- | --- | --- | --- | --- |
|  | Myoton: frequency | Myoton: stiffness | Myoton: decrement ^a^ | Nine-Hole Peg test | PPT: Deltoideus | PPT: ECRB | aRoM: extension | aRoM: flexion | aRoM: total | Isometric strength |
| Myoton: frequency | - | **0.875**  **(<0.001)** | 0.190  (0.315) | -0.312  (0.093) | 0.216  (0.252) | -0.039  (0.836) | -0.168  (0.376) | -0.21  (0.265) | -0.242  (0.197) | 0.462  (0.01) |
| Myoton: stiffness | - | - | **0.572**  **(0.001)** | -0.310  (0.095) | 0.067  (0.723) | -0.143  (0.450) | -0.105  (0.583) | 0.047  (0.806) | -0.016  (0.933) | 0.208  (0.271) |
| Myoton: decrement | - | - | - | -0.076  (0.690) | -0.167  (0.377) | -0.312  (0.093) | 0.163  (0.39) | 0.518  (0.003) | 0.485  (0.007) | -0.458  (0.011) |
| Nine-Hole Peg test | - | - | - | - | -0.159  (0.401) | -0.104 (0.584) | -0.187  (0.322) | -0.041  (0.828) | -0.134  (0.479) | 0.008  (0.968) |
| PPT: Deltoideus | - | - | - | - | - | **0.644**  **(<0.001)** | -0.152  (0.423) | -0.141  (0.456) | -0.193  (0.308) | 0.348  (0.06) |
| PPT: ECRB | - | - | - | - | - | - | -0.12  (0.529) | -0.151  (0.427) | -0.191  (0.312) | 0.365  (0.047) |
| aRoM: extension | - | - | - | - | - | - | - | 0.051  (0.787) | **0.674**  **(<0.001)** | -0.305  (0.101) |
| aRoM: flexion | - | - | - | - | - | - | - | - | **0.770**  **(<0.001)** | **-0.585**  **(0.001)** |
| aRoM: total | - | - | - | - | - | - | - | - | - | **-0.612**  **(<0.001)** |
| Isometric strength | - | - | - | - | - | - | - | - | - | - |
| N=30. Values are represented as Pearsons's ρ (level of significance). aRoM: active range of motion; ECRB: extensor carpi radialis brevis; PPT: Pressure Pain Threshold. Values in bold represent a significant correlation after correction for multiple comparisons (P<0.006). | | | | | | | | | | |

| **S-Table 3.** Correlation values and significance levels between different test assessing the wrist extensors muscles at the non-dominant side on assessment-1. | | | | | | | | | | |
| --- | --- | --- | --- | --- | --- | --- | --- | --- | --- | --- |
|  | Myoton: frequency | Myoton: stiffness | Myoton: decrement ^a^ | Nine-Hole Peg test | PPT: Deltoideus | PPT: ECRB | aRoM: extension | aRoM: flexion | aRoM: total | Isometric strength |
| Myoton: frequency | - | **0.907**  **(<0.001)** | -0.116  (0.543) | 0.132  (0.486) | -0.012  (0.949) | -0.128  (0.500) | -0.356  (0.053) | **-0.517**  **(0.003)** | **-0.574**  **(0.001)** | 0.384  (0.036) |
| Myoton: stiffness | - | - | 0.206  (0.274) | 0.119  (0.533) | -0.069  (0.717) | -0.194  (0.304) | -0.304  (0.102) | -0.314  (0.091) | -0.403  (0.027) | 0.234  (0.214) |
| Myoton: decrement | - | - | - | 0.024  (0.899) | -0.078  (0.683) | -0.114  (0.548) | -0.057  (0.764) | 0.367  (0.046) | 0.205  (0.276) | -0.484  (0.007) |
| Nine-Hole Peg test | - | - | - | - | 0.077  (0.687) | 0.107  (0.573) | -0.185  (0.329) | -0.001  (0.998) | -0.109  (0.567) | -0.043  (0.822) |
| PPT: Deltoideus | - | - | - | - | - | **0.839**  **(<0.001)** | 0.071  (0.708) | 0.079  (0.68) | 0.097  (0.611) | 0.198  (0.295) |
| PPT: ECRB | - | - | - | - | - | - | 0.190  (0.314) | 0.165  (0.384) | 0.223  (0.237) | 0.239  (0.203) |
| aRoM: extension | - | - | - | - | - | - | - | 0.199  (0.291) | **0.745**  **(<0.001)** | -0.175  (0.354) |
| aRoM: flexion | - | - | - | - | - | - | - | - | **0.800**  **(<0.001)** | -0.323  0.081 |
| aRoM: total | - | - | - | - | - | - | - | - | - | -0.324  0.081 |
| Isometric strength | - | - | - | - | - | - | - | - | - | - |
| N=30. Values are represented as Pearsons's ρ (level of significance). aRoM: active range of motion; ECRB: extensor carpi radialis brevis; PPT: Pressure Pain Threshold. Values in bold represent a significant correlation after correction for multiple comparisons (P<0.006). | | | | | | | | | | |

| **S-Table 4.** Correlation values and significance levels between different test assessing the wrist extensors muscles at the non-dominant side on assessment-2. | | | | | | | | | | |
| --- | --- | --- | --- | --- | --- | --- | --- | --- | --- | --- |
|  | Myoton: frequency | Myoton: stiffness | Myoton: decrement ^a^ | Nine-Hole Peg test | PPT: Deltoideus | PPT: ECRB | aRoM: extension | aRoM: flexion | aRoM: total | Isometric strength |
| Myoton: frequency | - | **0.901**  **(<0.001)** | 0.043  (0.820) | 0.021  (0.913) | 0.103  (0.588) | -0.076  (0.690) | -0.255  (0.173) | -0.367  (0.046) | -0.415  (0.023) | 0.412  (0.024) |
| Myoton: stiffness | - | - | 0.388  (0.034) | -0.036  (0.852) | 0.074  (0.698) | -0.159  (0.400) | -0.190  (0.314) | -0.159  (0.400) | -0.231  (0.220) | 0.265  (0.157) |
| Myoton: decrement | - | - | - | -0.247  (0.188) | -0.033  (0.863) | -0.176  (0.351) | -0.010  (0.959) | 0.451  (0.012) | 0.306  (0.100) | -0.418  (0.022) |
| Nine-Hole Peg test | - | - | - | - | -0.312  (0.093) | -0.202  (0.285) | 0.011  (0.954) | -0.085  (0.656) | -0.050  (0.794) | -0.015  (0.936) |
| PPT: Deltoideus | - | - | - | - | - | **0.788**  **(<0.001)** | 0.023  (0.905) | -0.052  (0.785) | -0.015  (0.938) | 0.390  (0.033) |
| PPT: ECRB | - | - | - | - | - | - | 0.113  (0.552) | 0.160  (0.398) | 0.193  (0.306) | 0.324  (0.080) |
| aRoM: extension | - | - | - | - | - | - | - | 0.115  (0.546) | **0.708**  **(<0.001)** | -0.310  (0.095) |
| aRoM: flexion | - | - | - | - | - | - | - | - | **0.781**  **(<0.001)** | -0.376  0.040 |
| aRoM: total | - | - | - | - | - | - | - | - | - | -0.449  0.013 |
| Isometric strength | - | - | - | - | - | - | - | - | - | - |
| N=30. Values are represented as Pearsons's ρ (level of significance). aRoM: active range of motion; ECRB: extensor carpi radialis brevis; PPT: Pressure Pain Threshold. Values in bold represent a significant correlation after correction for multiple comparisons (P<0.006). | | | | | | | | | | |

| **S-Table 5.** Correlation values and significance levels between different test assessing the wrist extensors muscles asymmetries on assessment-1. | | | | | | | | | | |
| --- | --- | --- | --- | --- | --- | --- | --- | --- | --- | --- |
|  | Myoton: frequency | Myoton: stiffness | Myoton: decrement ^a^ | Nine-Hole Peg test | PPT: Deltoideus | PPT: ECRB | aRoM: extension | aRoM: flexion | aRoM: total | Isometric strength |
| Myoton: frequency | - | **0.919**  **(<0.001)** | 0.335  (0.071) | 0.250  (0.183) | 0.100  (0.600) | 0.082  (0.667) | -0.126  (0.506) | -0.036  (0.85) | -0.128  (0.502) | -0.026  (0.893) |
| Myoton: stiffness | - |  | 0.468  (0.009) | 0.222  (0.239) | 0.101  (0.594) | -0.109  (0.566) | -0.133  (0.482) | 0.014  (0.94) | -0.089  (0.64) | -0.125  (0.51) |
| Myoton: decrement | - |  |  | 0.017  (0.928) | 0.412  (0.024) | -0.272  (0.146) | -0.164  (0.388) | 0.016  (0.933) | -0.118  (0.533) | -0.376  (0.04) |
| Nine-Hole Peg test | - |  |  |  | 0.100  (0.601) | 0.258  (0.169) | 0.017  (0.93) | -0.010  (0.959) | 0.029  (0.88) | 0.016  (0.932) |
| PPT: Deltoideus | - |  |  |  |  | 0.093  (0.624) | -0.284  (0.128) | 0.218  (0.247) | -0.045  (0.815) | -0.156  (0.411) |
| PPT: ECRB | - |  |  |  |  |  | 0.030  (0.875) | 0.115  (0.544) | 0.105  (0.58) | 0.087  (0.647) |
| aRoM: extension | - |  |  |  |  |  |  | -0.028  (0.885) | **0.633**  **(<0.001)** | 0.294  (0.115) |
| aRoM: flexion | - |  |  |  |  |  |  |  | **0.747**  **(<0.001)** | -0.050  0.792 |
| aRoM: total | - |  |  |  |  |  |  |  |  | 0.121  0.524 |
| Isometric strength | - | - | - | - | - | - | - | - | - | - |
| N=30. Values are represented as Pearsons's ρ (level of significance). aRoM: active range of motion; ECRB: extensor carpi radialis brevis; PPT: Pressure Pain Threshold. Values in bold represent a significant correlation after correction for multiple comparisons (P<0.006). | | | | | | | | | | |

| **S-Table 6.** Correlation values and significance levels between different test assessing the wrist extensors muscles asymmetries on assessment-2. | | | | | | | | | | |
| --- | --- | --- | --- | --- | --- | --- | --- | --- | --- | --- |
|  | Myoton: frequency | Myoton: stiffness | Myoton: decrement ^a^ | Nine-Hole Peg test | PPT: Deltoideus | PPT: ECRB | aRoM: extension | aRoM: flexion | aRoM: total | Isometric strength |
| Myoton: frequency | - | **0.919**  **(<0.001)** | 0.040  (0.832) | 0.213  (0.258) | -0.188  (0.320) | -0.226  (0.230) | -0.302  (0.105) | -0.087  (0.646) | -0.325  (0.080) | -0.074  (0.699) |
| Myoton: stiffness | - |  | 0.351  (0.057) | 0.154  (0.418) | -0.084  (0.660) | -0.278  (0.137) | -0.302  (0.105) | 0.004  (0.981) | -0.265  (0.157) | 0.043  (0.820) |
| Myoton: decrement | - |  |  | -0.239  (0.204) | 0.158  (0.405) | -0.255  (0.173) | -0.014  (0.941) | 0.280  (0.133) | 0.151  (0.427) | 0.218  (0.248) |
| Nine-Hole Peg test | - |  |  |  | -0.058  (0.762) | 0.110  (0.564) | 0.204  (0.281) | -0.189  (0.316) | 0.083  (0.665) | -0.083  (0.663) |
| PPT: Deltoideus | - |  |  |  |  | 0.186  (0.326) | 0.087  (0.647) | 0.068  (0.721) | 0.192  (0.31) | 0.326  (0.079) |
| PPT: ECRB | - |  |  |  |  |  | 0.165  (0.382) | -0.205  (0.276) | 0.042  (0.824) | 0.160  (0.399) |
| aRoM: extension | - |  |  |  |  |  |  | -0.232  (0.217) | **0.715**  **(<0.001)** | 0.355  (0.054) |
| aRoM: flexion | - |  |  |  |  |  |  |  | **0.491**  **0.006** | -0.012  0.951 |
| aRoM: total | - |  |  |  |  |  |  |  |  | 0.302  0.104 |
| Isometric strength | - | - | - | - | - | - | - | - | - | - |
| N=30. Values are represented as Pearsons's ρ (level of significance). aRoM: active range of motion; ECRB: extensor carpi radialis brevis; PPT: Pressure Pain Threshold. Values in bold represent a significant correlation after correction for multiple comparisons (P<0.006). | | | | | | | | | | |
